# Applying Community Engagement Methods to Facilitate Global Co‑Learning among Indigenous Communities

**DOI:** 10.5334/aogh.5057

**Published:** 2026-04-17

**Authors:** Sonya Shin, Nancy Rumaldo, Carmen George, Karen Ramos Carhuas, Genaro Anco, Malyssa Egge, Louise Benally, Hilda Quispe, Kerlissa Bitah, Anglene Joe, Casey Dai, Jioni Tuck, Jesus Peinado

**Affiliations:** 1 Division of Global Health Equity, Brigham and Women’s Hospital, Boston MA, USA; 2 Department of Global Health and Social Medicine, Harvard Medical School, Boston MA, USA; 3 Socios En Salud, Lima, Peru; 4 Community member, Navajo Nation, USA; 5 Navajo Nation Head Start Program, St Michaels AZ, USA; 6 Harvard School of Public Health, Boston MA, USA; 7 Community member, Maras, Cusco, Peru

**Keywords:** community engagement, Navajo, indigenous, Quechua, Peru, climate change, water, child health, global learning, global health

## Abstract

Principles and methods for community engagement in research align closely with the Global Learning for Health Equity Framework. Both concepts center around trust, respect, reciprocity, and humility; elevate the inherent wisdom of community perspectives; and emphasize opportunities for equitable, bidirectional learning and co-creation across the project’s life cycle. Our team utilized community engagement methods in a global learning initiative, engaging two Indigenous communities in Peru and Navajo Nation. In the initial year of this project, we used both cross-site and site-specific community engagement methods with the collective goal of identifying local strategies to address water insecurity and advance child health. In this viewpoint, we describe the community engagement process and reflect on challenges, lessons learned, and future directions.

## Introduction

Community engagement in research (CENR) is an active participatory process which allows people to define and address issues affecting their community through inclusive and authentic partnership [1, 2]. CENR overlaps with various concepts, including knowledge co‑production [3], community‑based participatory research [4], and participatory action research [5]. While the level and forms of community engagement are highly dependent on context and relationships [6], the CENR literature consistently identifies core principles: sharing power, building trusting and transparent relationships, creating systems for equitable inclusion of community members, and ensuring reciprocity and mutual benefit [3, 4, 7, 8]. By ensuring that research priorities are community‑driven and adapted to local settings and culture, CENR results in greater impact and uptake of study findings [9]. CENR can be radically transformative by shifting power to those most impacted and marginalized, while uninformed or superficial attempts at CENR can further entrench disparities between community groups and researchers or among community members themselves. Examples of pitfalls include limited decision‑making power, skewed selection of community representatives, expecting reciprocity or imposing roles, and over‑promising or failing to address divergent priorities [10–12].

**Figure 1:**
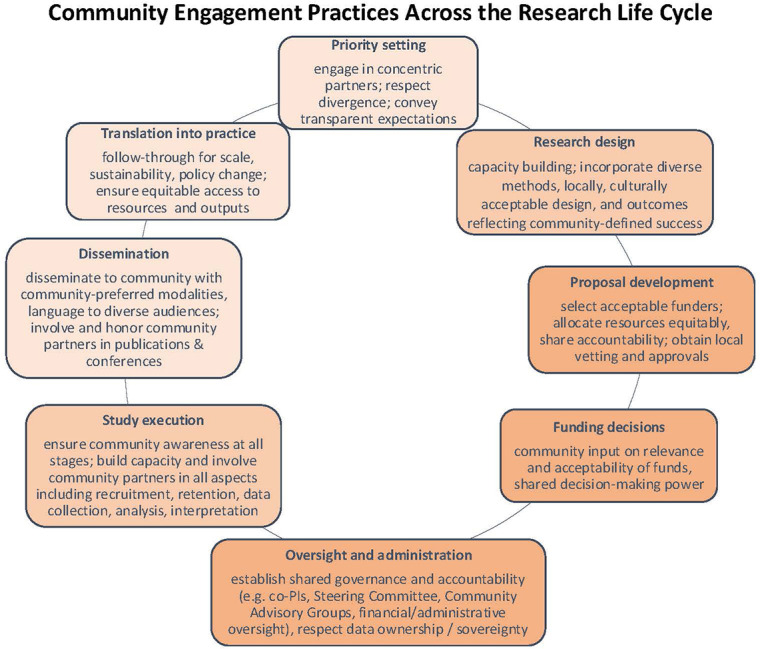
Adapted with permission from Tembo D, Hickey G, Montenegro C, et al. Effective engagement and involvement with community stakeholders in the co‑production of global health research. BMJ. 2021;372, licensed under CC BY‑NC 3.0 IGO (https://creativecommons.org/licenses/by-nc/3.0/igo/).

CENR is conceptualized across a continuum of power sharing [4, 7, 8]. Sanders Thompson et al. describe the relative contributions of researchers and community members, with community partners gaining power and decision‑making as community engagement increases [5]. This process takes time. In fact, the highest level of engagement—partnerships—transcends individual projects and is predicated on pre‑existing trust and strong relationships [5]. For this reason, many authors argue that CENR requires investing in “partnerships not projects” so that researchers can truly apply CENR at all stages of the research life cycle [11] (Figure 1). This includes “starting early” with priority setting [13–15] and “sticking around” to ensure local impact after a study is done [6, 7]. While Figure 1 refers specifically to research, the CENR framework can arguably apply to the life cycle of any community‑based project.

CENR principles are closely aligned with core values described by the Global Learning for Health Equity (GL4HE) framework: trust, respect, reciprocity, and humility [16]. The GL4HE framework is designed as an implementation tool for US practitioners interested in seeking health solutions outside the USA for adaptation and implementation in their own local communities. Funded by the Robert Wood Johnson Foundation and led by a consortium of implementers, researchers, and community leaders, the GL4HE Network strives to promote global learning as an emerging and relevant discipline in health equity research [16, 17]. While global learning may reflect varying degrees of community engagement among US and global partners [18], the GL4HE framework emphasizes the critical role of community engagement and bidirectional community‑to‑community learning to achieve both equitable knowledge sharing and equitable health outcomes [16].

Given the robust literature on CENR best practices, understanding how specific CENR methodologies can be incorporated into GL4HE is needed. This manuscript describes an initiative incorporating CENR methods in an initial priority‑setting phase of GL4HE which brought together new community partners from two Indigenous communities. The activities were undertaken by a triad of collaborators—an academic research team, community partners from Navajo Nation, and community partners from Peru—to identify community‑led solutions and shared priority setting across these communities. The goal of this article is to describe both challenges and lessons learned in a year‑long GL4HE initiative using community engagement methods to identify priorities to address health challenges of water insecurity experienced by two Indigenous communities.

## Impetus for Indigenous Global Learning for Health Equity

In 2009, a researcher (SSS) with a 15‑year partnership with a non‑profit organization in Peru (Socios En Salud / Partners In Health) began a community health program on Navajo Nation, applying global health models to improve health outcomes among Navajo patients and families [19]. Over the ensuing decade, her work at both sites in early child health evolved along separate parallel paths guided by community priorities and local health disparities. In both sites, teams had recently completed research documenting the challenges of water insecurity and its effects on maternal and child health, which had been disseminated back to respective communities [20, 21]. Share‑back discussions revealed interest in both communities to more deeply understand and address water insecurity in the broader context of climate change, presenting the opportunity for a new future cycle of research and implementation (Figure 1). Up to this point, community discussions of these themes had been parallel and separate. However, the team’s involvement in the GL4HE Network prompted us to consider how we could bring together Navajo and Quechua community partners from both sites to pursue convergent dialogue using intentional CENR methods to foment bidirectional learning and consensus priority setting on a salient topic for both sites: water insecurity and its impact on child health.

The liaison researcher invited a research colleague from each site (NR, CG) to co‑lead the project. We speculated that perspectives from two Indigenous communities would be specific to place and culture, yet share common understandings and solutions. This premise was grounded in the understanding that health disparities experienced by Indigenous people are deeply rooted in colonizing practices of globalization, including economic and social exclusion, disruption of cultural and linguistic preservation, and environmental infractions by governmental and industry actors [22, 23]. At the same time, protective factors of Indigenous communities—including self‑determination and cultural continuity—are protective across a spectrum of health conditions [24, 25]. By bringing together diverse Indigenous communities, we sought to identify strategies addressing water insecurity that would be locally applicable yet potentially generalizable or adaptable across global Indigenous settings.

We utilized CENR methods and applied the principles put forth by Deivanayagam et al., which posit that solutions start by acknowledging the impact of environmental injustices and work to shift hierarchies of power, epistemologies, and decision‑making [26]. Inherent in this process was recognizing the power hierarchies inherent to academic‑community and US‑international partnerships and taking active measures to shift decision‑making to local community members. Taking these dynamics into account and our emphasis on open‑ended listening, this project did not constitute research which seeks to develop generalizable knowledge. Rather, the goal of this project was to create spaces for community dialogue and build new bridges with community members as peer experts, as opposed to study participants. We shared the transparent goal of generating community‑owned reflections with the hope of leading to future collaborations, including the potential for research if deemed relevant. The project took place over a 12‑month period, including a cross‑site convening and a series of community listening sessions at each site. Individuals who took part in local community listening sessions received compensation for their time and expertise.

## Cross‑Site Convening

Funded by Harvard Radcliffe Institute, we hosted a convening on Indigenous Learning in Water Security and Early Child Health in April 2024 in Boston, MA. Inviting community members from Peru and Navajo Nation, we sought to explore the intersection of water security and climate change as a threat to early child health from an Indigenous perspective. Both sites had community‑based early child health programs and expressed interest in future inter‑disciplinary, cross‑cultural intervention research on water security and climate change. Thus, both sites had mature community partnerships and were ready to embark on planning future projects. The goal of the event was to identify shared solutions and ideas to address the intersection of water and early child health.

The Radcliffe convening and follow‑up community listening process reflected intentional efforts to incorporate community engagement throughout this initial phase of sense‑making and priority setting. While the liaison researcher had long‑standing collaborations with both communities, participants from Peru and Navajo Nation had not previously collaborated or interacted. Creating a safe space to build trust was a primary goal, of equal importance to idea generation. Here, we describe some of the core aspects of planning, execution, and follow‑up, including challenges and lessons learned of community engagement using a racial justice approach.

*Participant selection:* We sought to include Peruvian and Diné (Navajo) participants who represented cross‑sector experience, a diversity of lived experiences, and local leadership roles. The 10 participants included the liaison researcher and 9 community members (4 Peruvians, 5 Diné), of whom 6 were Indigenous (4 Diné, 2 Quechua). Participation barriers primarily affected Peruvians: obtaining passports and visas and ensuring the ability to cover up‑front expenses represented meaningful systemic barriers that differentially impacted international participants, even more so, those from remote rural communities.

*Building trust and honoring culture:* We secured additional funds to invite three Peruvians to take part in a three‑day visit to Navajo Nation, in advance of the convening. This prelude allowed Peruvian visitors to build friendships and gain first‑hand exposure to Diné culture, community‑based strengths and challenges related to water security and climate change. For the convening, we modified our agenda to accommodate the solar eclipse which took place on the afternoon of Day 1. In observance of Diné culture, the meeting was adjourned during the eclipse and resumed in the evening. A Diné traditional knowledge holder invited all participants to offer a corn pollen blessing after the eclipse and to greet the ocean sunrise the following day with prayer (Picture 1). These offerings allowed the team to build a stronger bond and set shared intentions for the convening. The generosity of participants in sharing about their culture and trusting others was essential for the convening’s success.

**Picture 1:**
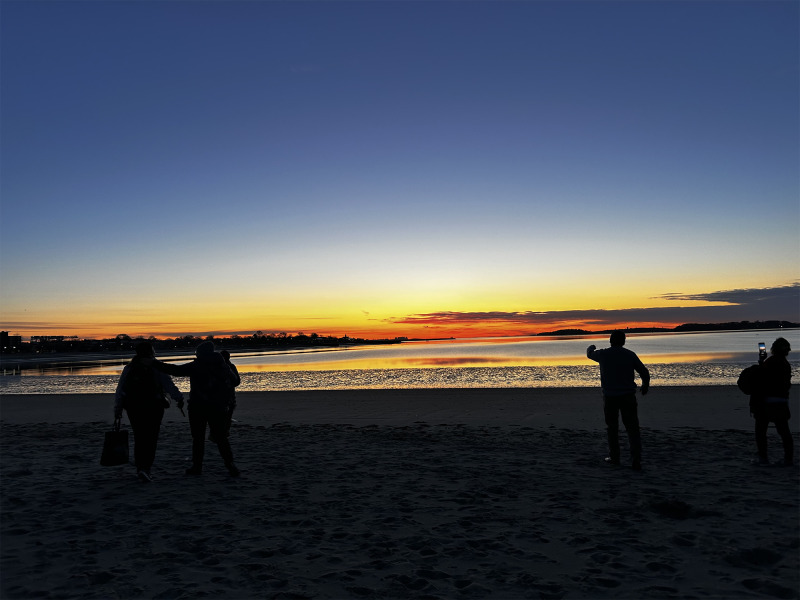
Sunrise greeting at cross‑site convening.

The convening sought to empower Indigenous participants by minimizing power hierarchies inherent to academic‑community dialogue, centering expertise in the lived experiences of community members, and making efforts to build trust and exchange cultural knowledge. For example, facilitation was carried out by community members and bilingual students, rather than the liaison researcher. Despite these efforts, the group did experience subtle challenges in equity related to language. At times, translation was time‑consuming to ensure that everyone fully understood the conversation, and despite translator support, dialogue was English‑centered. Very rarely did individuals speak Diné or Quechua languages, reflecting dominant hierarchies. Another limitation of the convening was the *a priori* focus on water insecurity and climate change, rather than a truly open conversation related to all manifestations of climate change. While this focus reflected ongoing work and local priorities at both sites and helped to advance the discussion, the focused discussion may have pruned the full spectrum of community perspectives, including other manifestations of climate change impact on health.

*Generating and exchanging knowledge:* The event was organized to ensure that perspectives of community members were central to the generative discussion and learning process. Printed materials were prepared and shared to quickly establish foundational knowledge about climate change science (Appendix 1) and contextual information about water security, climate change, and health in both sites as infographics. The seminar then transitioned to a gallery walk of photos and presentations by community members (Picture 2). Participants were invited to bring a photo or two that illustrated the importance of water in their community (Appendix 2). Facilitators encouraged dialogue around common themes, serving as a foundation on Day 1 to identify key problems and community strengths. Navajo Nation partners described the spiritual importance of water, as well as concerns about water contamination with heavy metals. They highlighted a project in which youth conducted water testing to understand the health of water in their own communities. The Peru‑based team profiled three communities: the rural Maras District of Peru where water scarcity has increased, causing challenges with agricultural production; rural Arequipa where communities have worked to restore and protect water sources through nature‑based solutions and organizing against mining companies; and urban Lima where migration has led to new communities in need of water infrastructure.

**Picture 2:**
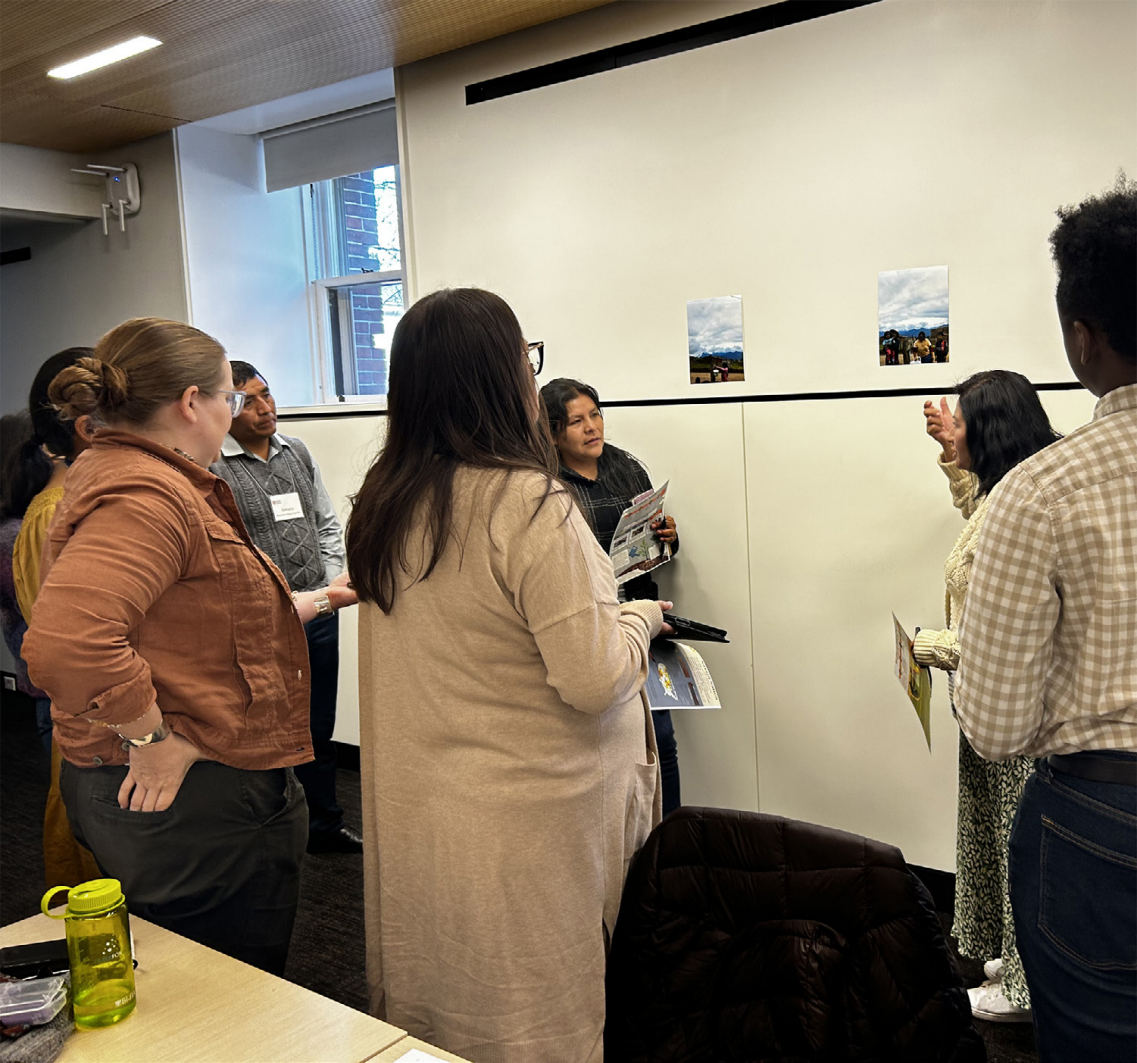
Gallery walk to promote cross‑site learning.

## Finding Common Ground

The second day delved into problem‑solving, facilitating small‑group discussion to allow each site to generate solutions and share back to the larger group for reflections. Facilitators guided discussion toward convergence around common solutions, i.e., solutions independently proposed by both sites and solutions identified by one site to which the other site agreed. While implementation details and concepts varied across sites, the group arrived at six core solutions: water and land conservation, educating children and families about water conservation and environmental stewardship, community engagement and organizing, supporting families and neighborhoods facing water insecurity, strengthening health care systems for future climate change threats, and intergenerational transmission of cultural knowledge related to water. All participants agreed upon the importance of collaboration among Indigenous communities to address shared challenges. They also emphasized the need for financial and educational support to empower local communities to solve local water security issues.

## Community Listening Sessions

Based upon the core solutions identified at the convening, each group developed a plan and managed a small budget ($5000 per site) to engage a broader sphere of local community members for feedback on potential solutions. This process of “concentric community consultation” starts with a small group and expands to deeper community consultation with more representative constituents to explore community readiness, ongoing efforts, and priority setting of strategies most relevant for local adaptation. Each site identified stakeholders and a process for community listening. In Peru, we organized a two‑day workshop with Maras community leaders (n = 14) and a single‑day workshop in Lima (n = 9). In Navajo Nation, we worked with regional community leaders to organize two‑hour listening sessions in five communities (n = 68), followed by a share‑back session with each community.

Out of this process, a set of strategies has been co‑developed with community partners, with whom we are currently working to develop proposals to implement and evaluate these interventions. In Navajo Nation, the core strategies have coalesced around water and land conservation, educating children and families on environmental and water stewardship, gathering intergenerational stories about local water sources, and piloting a water voucher program for households with young children. In Maras, top strategies include increasing household access to potable water and implementing nature‑based solutions such as propagating native plant species known to attract water, restoring natural reservoirs using pre‑Incan technology, and improving irrigation infrastructure to enhance efficiency of agricultural water use. While each site had very different solutions, they converged around water conservation efforts grounded in long‑standing Indigenous practices and culture, and providing resources for families and communities experiencing water insecurity.

## Conclusion

Community engagement is an iterative process that takes time and intentionality to tackle the underlying power structures that are inherent to cross‑site academic‑community or community‑community partnerships. By focusing our collaboration on trust building, sharing of culture, and equitable dialogue, we successfully gathered community‑based solutions to address climate change, water insecurity, and child health in diverse Indigenous communities. Through concentric community consultation, each site arrived at local solutions prioritized by community partners while retaining shared core strategies and long‑term goals. Future plans involve selecting community strategies which align with our team’s research focus, identifying additional partners to address expertise gaps, and co‑creating proposals with research goals that are relevant to community partners. In Navajo Nation, we are exploring multilevel interventions to increase water security and promote climate resilience strategies through intergenerational community outreach. In Maras, we have expanded our partnership to explore multilevel strategies to increase household water security and implement nature‑based solutions. In addition, by harmonizing outcomes and causal models, we seek to link these projects to continue shared learning and increase the promise of creating generalized knowledge.

Box 1 summarizes key lessons learned. Challenges also emerged, including structural inequities in the “mundane” details of travel and translation. Also, despite the intention to shift decision‑making from academic to community actors, input from the research team—including the liaison researcher—was still needed to advance the project (e.g., analyzing data, effectively sharing back information to community partners, building partnerships, and fundraising). We also navigated the inherent tension of two agendas: shared global learning and community‑led solutions. On the one hand, we narrowed the discussion to water issues from the outset; on the other hand, community listening allowed priority setting by community partners without insisting on a cross‑site “shared strategy.” Nonetheless, the emergence of common themes and similar solutions indicate potential for synergy and generalizable solutions.

**Box 1: Lessons Learned**

- **Intentional selection of a framework and community engagement tools allowed the team to meet the goals of global learning for health equity.** *We applied CENR strategies and the GL4HE framework to the priority‑setting phase of a project to explore bidirectional learning and consensus priority setting across two Indigenous communities, Navajo Nation, USA and Maras, Peru.*
- **Defining our desired impact and guiding dialogue toward convergence allowed us to arrive at shared core strategies.** *Our process began by setting a shared goal of identifying community‑led strategies that could be implemented to advance child health equity by addressing water insecurity. After building shared knowledge and trust, we mapped solutions to identify shared core principles and strategies.*
- **Community engagement process involved concentric community engagement in each site and across sites.** *An initial convening included 4–5 community partners from each site, followed by a series of community listening and share‑back sessions held in each community to get broader and deeper feedback on strategies identified at the convening.*
- **Community engagement takes time, particularly to build trust and find common ground.** *Over 12 months, we undertook priority‑setting activities, first building shared knowledge and understanding across sites and then undertaking a deeper community‑led exploration at each site. Building a strong foundation of community partnerships will allow us to advance future research collaborations at each site and continue global co‑learning.*

We look forward to learning how to intentionally center our work in the community voice and cross‑site learning across the remaining cycle of research and project implementation.

## Additional Files

The additional files for this article can be found as follows:

Supplementary Appendix 1. Water and Climate Change Graphic.

Supplementary Appendix 2. Gallery Walk of Peruvian and Navajo communities.
