# Supplementary material for: Applying Community Engagement Methods to Facilitate Global Co‑Learning among Indigenous Communities
Source: Ann Glob Health. 2026 Apr 17;92(1):36. doi: 10.5334/aogh.5057 (PMC13089358; doi:10.5334/aogh.5057)
Supplement: Supplementary Appendix 1. — Water and Climate Change Graphic. [file agh-92-1-5057-s1.pdf]

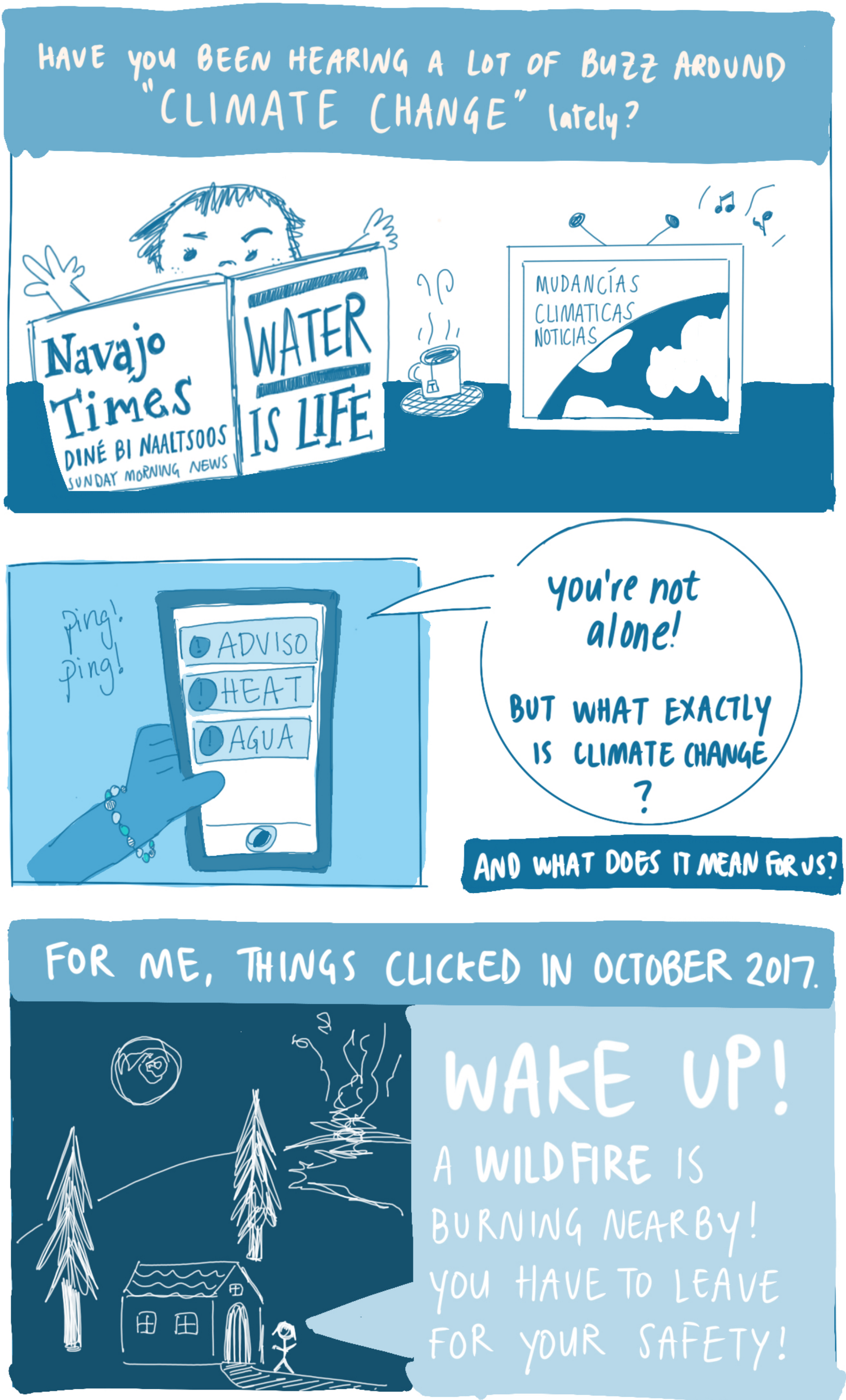
HAVE YOU BEEN HEARING A LOT OF BUZZ AROUND "CLIMATE CHANGE" lately?
Navajo Times
DINÉ BI NAALTSOOS
SUNDAY MORNING NEWS
WATER IS LIFE
MUDANCÍAS CLIMATICAS NOTICIAS
ping! ping!
ADVISO
HEAT
AGUA
you're not alone!
BUT WHAT EXACTLY IS CLIMATE CHANGE ?
AND WHAT DOES IT MEAN FOR US?
FOR ME, THINGS CLICKED IN OCTOBER 2017.
WAKE UP!
A WILDFIRE IS BURNING NEARBY! YOU HAVE TO LEAVE FOR YOUR SAFETY!

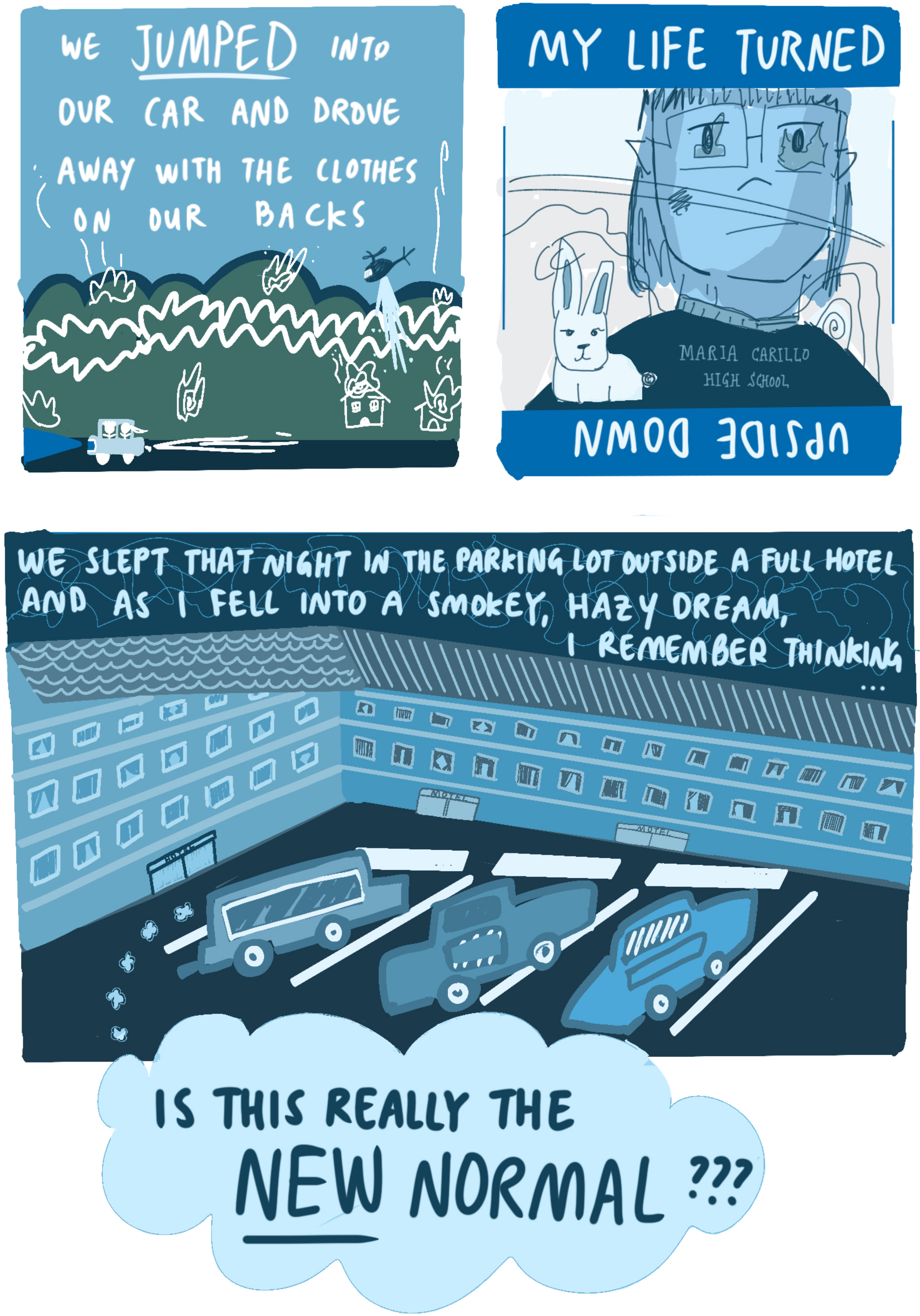
WE JUMPED INTO OUR CAR AND DROVE AWAY WITH THE CLOTHES ON OUR BACKS
MY LIFE TURNED
MARIA CARILLO HIGH SCHOOL
UPSIDE DOWN
WE SLEPT THAT NIGHT IN THE PARKING LOT OUTSIDE A FULL HOTEL AND AS I FELL INTO A SMOKEY, HAZY DREAM, I REMEMBER THINKING ...
MOTEL
MOTEL
IS THIS REALLY THE NEW NORMAL ???

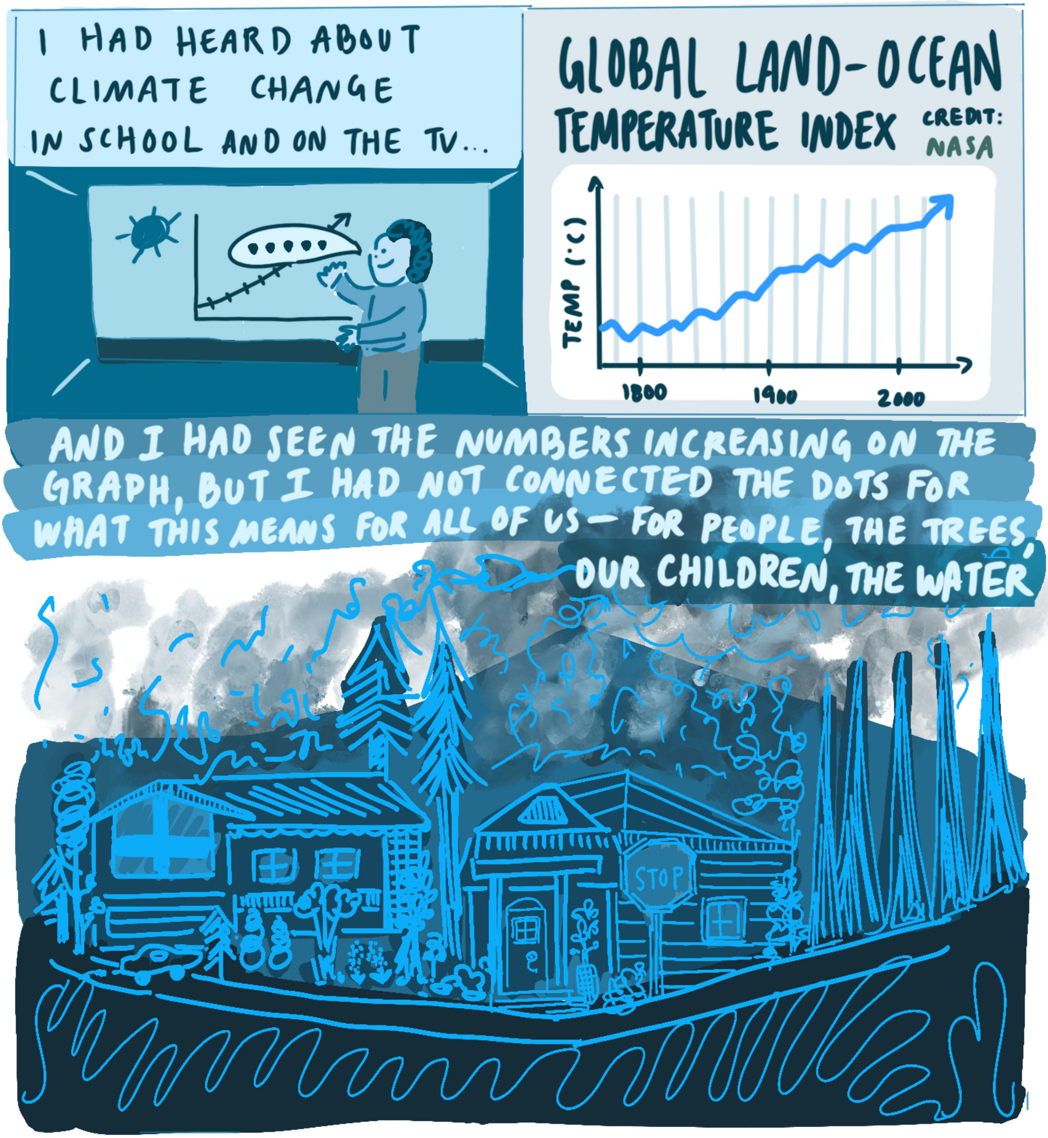

WILDFIRES ARE HAPPENING MORE OFTEN IN CALIFORNIA — AND THE PLACE I CALL HOME IS GETTING HOTTER AND DRIER IN THE SUMMER, AND WETTER IN THE WINTER, WHICH CAN MAKE WILDFIRES BIGGER AND MORE DEVESTATING.

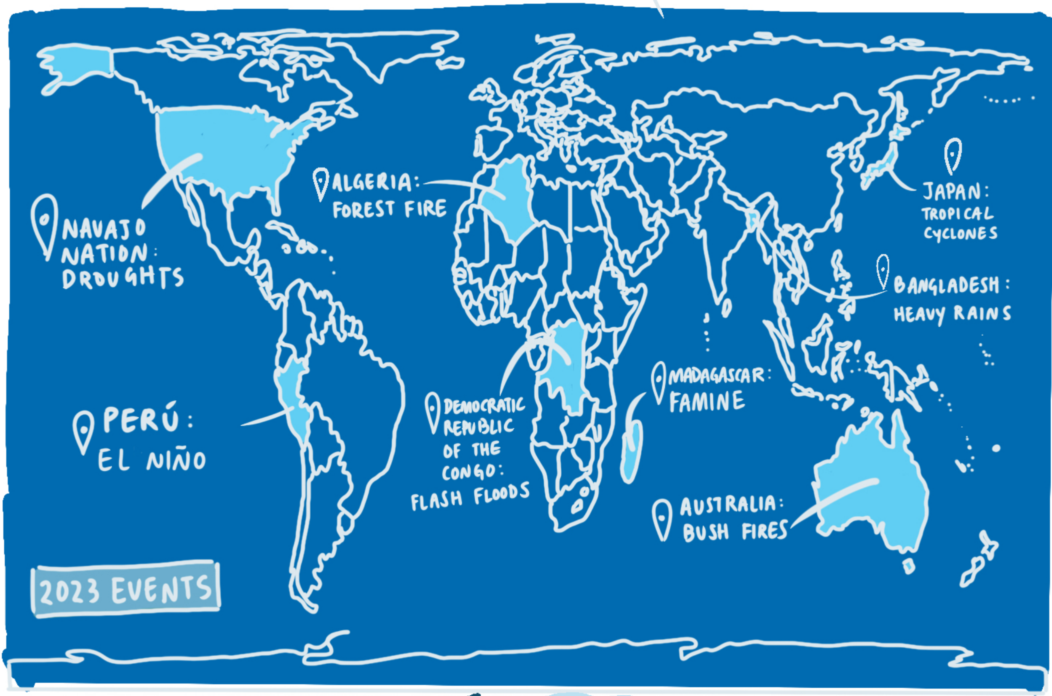

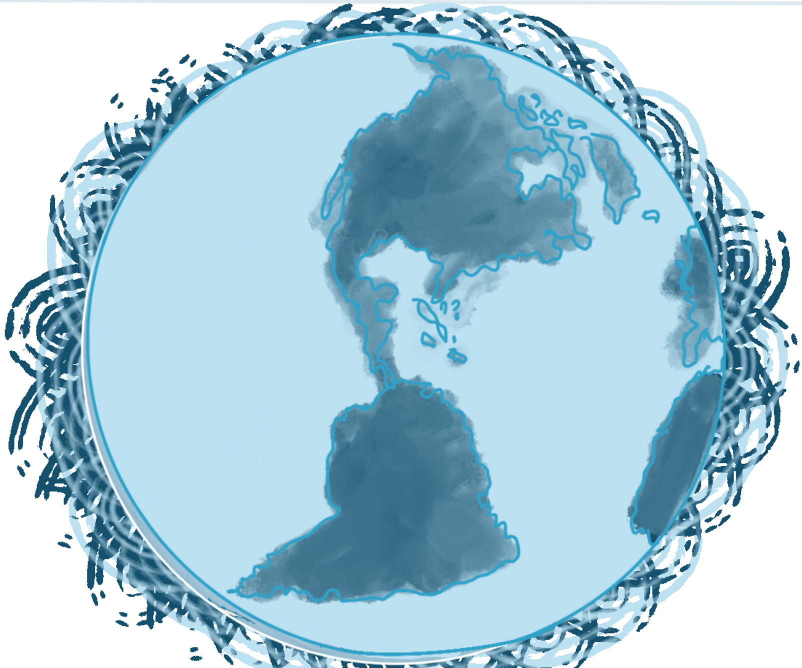

THE EARTH IS HEATING UP BECAUSE HUMANS—
LARGELY IN THE GLOBAL NORTH—
ARE CHANGING IT!

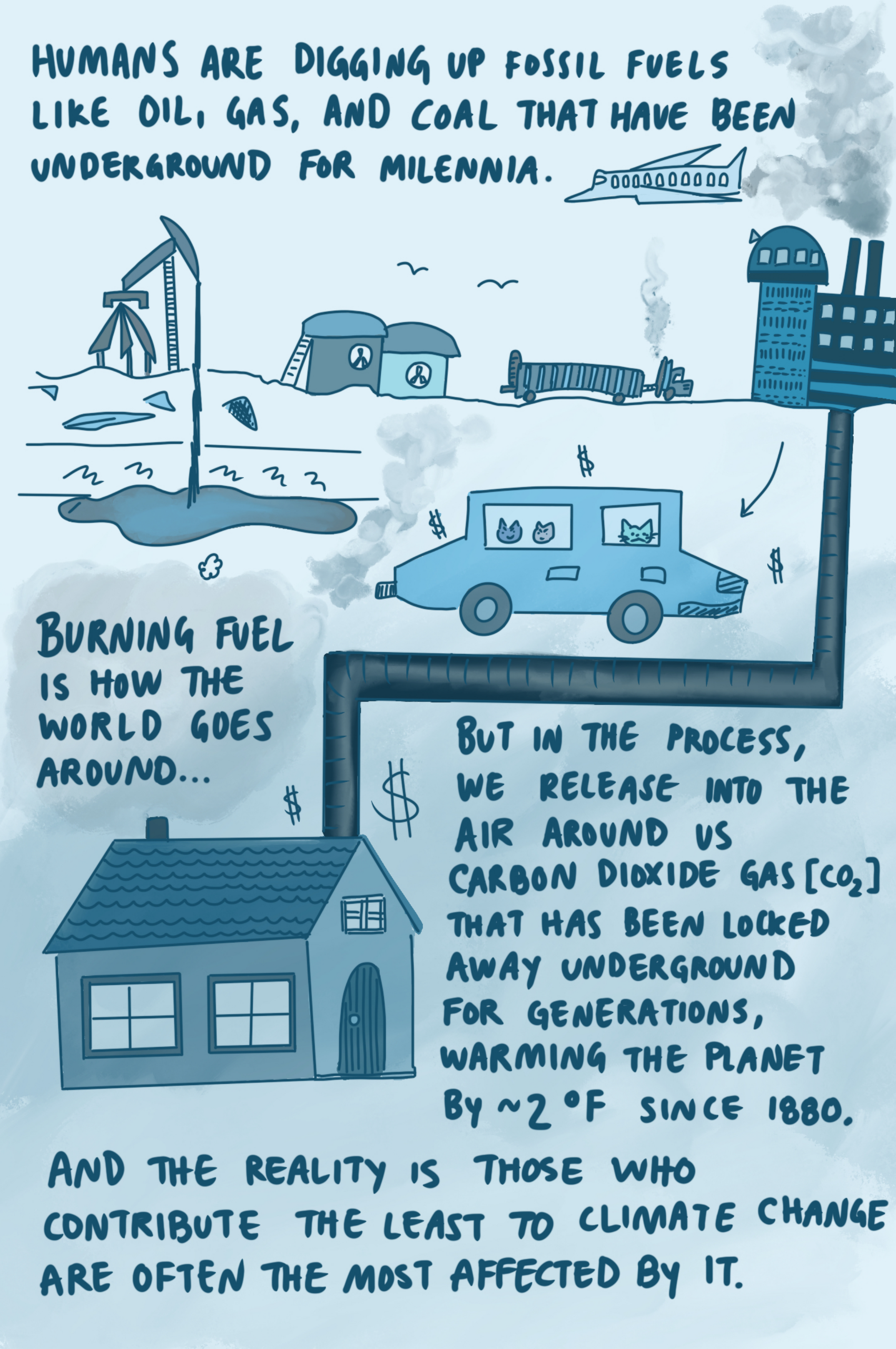
HUMANS ARE DIGGING UP FOSSIL FUELS LIKE OIL, GAS, AND COAL THAT HAVE BEEN UNDERGROUND FOR MILENNIA.
BURNING FUEL IS HOW THE WORLD GOES AROUND...
BUT IN THE PROCESS, WE RELEASE INTO THE AIR AROUND US CARBON DIOXIDE GAS [CO_2] THAT HAS BEEN LOCKED AWAY UNDERGROUND FOR GENERATIONS, WARMING THE PLANET BY ~2 °F SINCE 1880.
AND THE REALITY IS THOSE WHO CONTRIBUTE THE LEAST TO CLIMATE CHANGE ARE OFTEN THE MOST AFFECTED BY IT.

AS THE MERCURY RISES,
THE CLIMATE CHANGES.
FOR EVERY ~2°F INCREASE
IN GLOBAL AVERAGE TEMPERATURE,
SCIENTISTS PREDICT THAT
WATER SCARCITY WILL INCREASE, TOO.

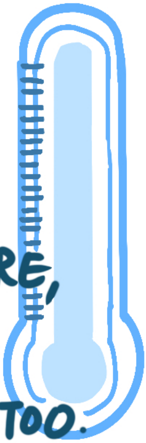

CLIMATE IMPACT	DOWNSTREAM EFFECTS
Decrease in rain: DROUGHT	↓ Reduction in drinking water supply<br>↓ Lower flow in rivers, streams<br>↑ Heavy metal, pesticide pollutants don't wash away and build up in waterways<br>↑ Disproportionate burden on children, who are more vulnerable than adults to climate stress<br>↳ Question for you: Why do you think this is so?

THIS ISN'T JUST A DROP
IN THE BUCKET —
WATER IS AN ESSENTIAL
HUMAN RIGHT.

WATER SCARCITY:

happens when communities cannot meet their water needs, either because water supplies are lacking, or because infrastructure is inadequate.
