# Supplementary material for: Applying Community Engagement Methods to Facilitate Global Co‑Learning among Indigenous Communities
Source: Ann Glob Health. 2026 Apr 17;92(1):36. doi: 10.5334/aogh.5057 (PMC13089358; doi:10.5334/aogh.5057)
Supplement: Supplementary Appendix 2. — Gallery Walk of Peruvian and Navajo communities. [file agh-92-1-5057-s2.pdf]

Appendix 2 Gallery Walk of Peruvian and Navajo communities

Four Corners Region, Navajo Nation

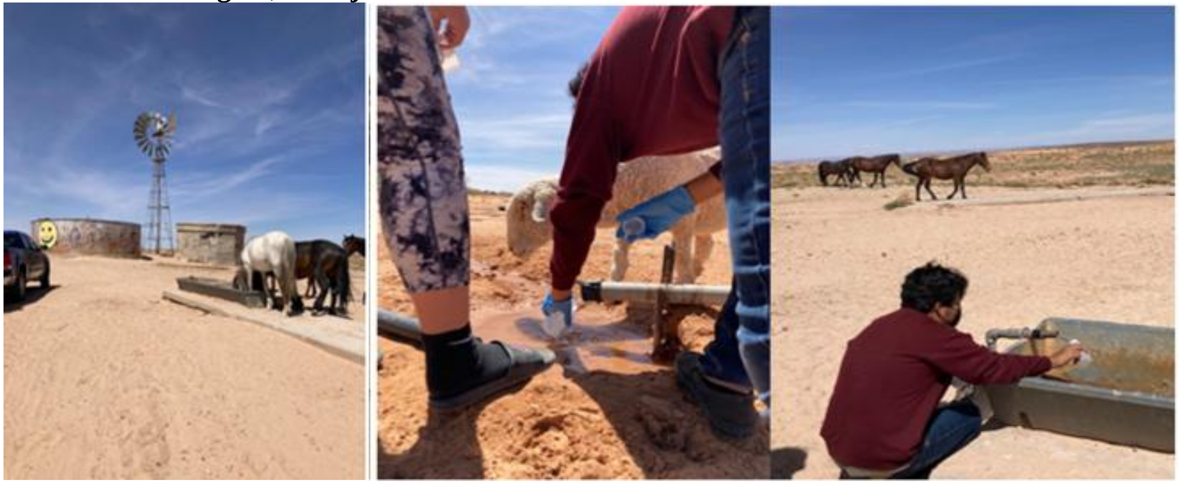

People, plants and animals rely on water, which is a precious resource. Water is alive. Like with any spirit or living being, we are taught to show respect when we speak about it, think about, and make plans or intentions that have to do with water. Our team interviewed elders and gathered their stories about water. Our team of community mentors and scientists worked with Diné youth who were able to choose what water they wanted to test in their own communities, collect the water and send it to an outside lab for testing. We collected water from the water sources including artesian wells. The wild horses know the local sources of water and would come over when we began to collect water. The youth were able to share back the results to their community members. We found that some of the unregulated water sources were contaminated with heavy metals. The drinking water sources all came back with good results, meaning uranium, arsenic, lead, petroleum levels were within EPA standard limits.

Maras District, Cusco, Peru

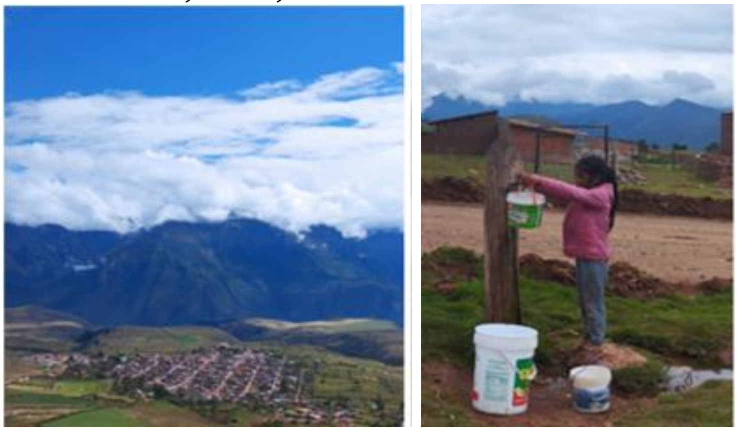

In Maras, we have seen that the water supply has gone down over the years. In the past, our mountain was covered in glacial snow, but that has started to disappear over the years, and now when you look at the mountain the snowcap is no longer there. The town of Maras has municipal water, but many of the smaller communities don't. Families have to go to the pump to collect water. Children have to carry these full containers back to their homes every day. Sometimes the pump has water but other times, it doesn't. We are also seeing that there is less and less water for our crops. The irrigation water has become less reliable, especially in the past few years due to drought. This has also caused problems because most families here rely on agriculture for their income and sustenance.

Huarhua Community, Arequipa, Peru

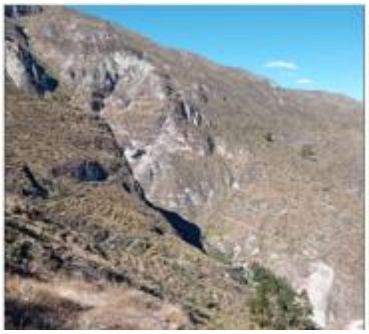
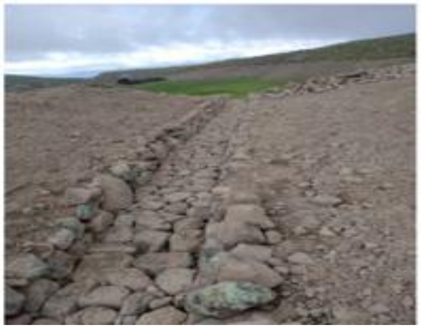
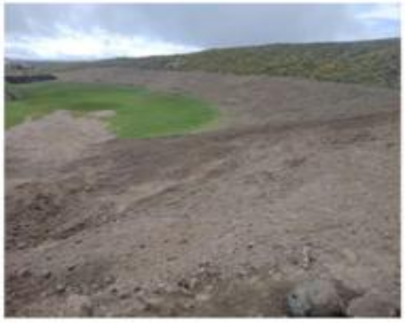

The most complicated time to move the water is during the rainy season. Many hills or Apus come down from the heights with stones and break the hoses and irrigation canals. For this reason, every year we have to replace the hoses with new ones. The consumption of water in my community is in a direct way without treatment and that often generates health problems in children, adults and expectant mothers. As solution strategies for water supply improvement: we are expanding the reservoirs where water is stored (ponds) to generate the volume of water for cultivation and consumption, we are cleaning the canals through which the water circulates until it reaches the communities, we have organized the communities in different places to denounce and not allow the mining companies to work since they have contaminated the springs from where the water filters and to stop contaminating since the life of human beings and animals is at risk in their habitat.

Carabayllo District, Lima, Peru

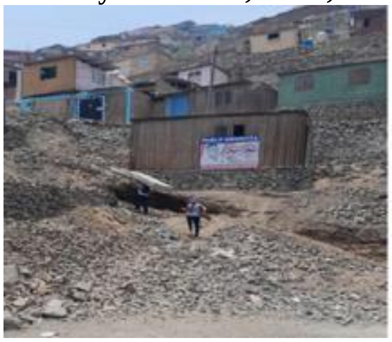
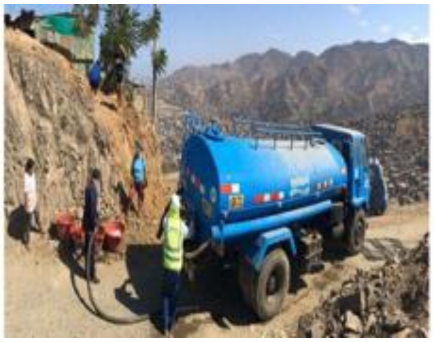
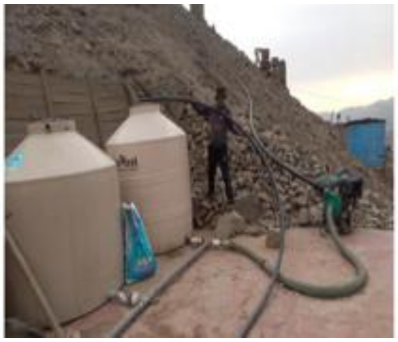

In this Asentamiento Humano, as we call it in Peru, we have approximately 5,500 inhabitants. We have limited access to drinking water, communication, education, quality food and housing, because many people migrate from other cities or communities to Lima in search of work and education. The distribution of water to the community is based on a mobile cistern that brings non-chlorinated water for sale on a weekly or biweekly basis and the residents buy it in a plastic bucket or tanks for storage. The cistern is a mobility that carries water to a certain point and from that place we must connect with a hose to the house, this hose can travel 1 to 2 kilometers. In my community of Carabayllo, we have organized with leaders of Asentamientos Humanos to request support from the local government to distribute water free of charge for a year, until we formally manage our community reservoir. The community is working on access (road) so that the water cistern can enter and the cost will be lower. The community is also organizing to request support from the central government for installation of soup kitchens to alleviate food expenses.
